# Variation Profile of the Orthotospovirus Genome

**DOI:** 10.3390/pathogens9070521

**Published:** 2020-06-29

**Authors:** Deepti Nigam, Hernan Garcia-Ruiz

**Affiliations:** Department of Plant Pathology and Nebraska Center for Virology, University of Nebraska-Lincoln, Lincoln, NE 68583, USA; deepti-nigam.singh@unl.edu

**Keywords:** intergenic region, single nucleotide polymorphism, RNA secondary structure, virus evolution, virus adaptation, thrips, insect vectors, TSWV

## Abstract

Orthotospoviruses are plant-infecting members of the family *Tospoviridae* (order *Bunyavirales*), have a broad host range and are vectored by polyphagous thrips in a circulative-propagative manner. Because diverse hosts and vectors impose heterogeneous selection constraints on viral genomes, the evolutionary arms races between hosts and their pathogens might be manifested as selection for rapid changes in key genes. These observations suggest that orthotospoviruses contain key genetic components that rapidly mutate to mediate host adaptation and vector transmission. Using complete genome sequences, we profiled genomic variation in orthotospoviruses. Results show that the three genomic segments contain hypervariable areas at homologous locations across species. Remarkably, the highest nucleotide variation mapped to the intergenic region of RNA segments S and M, which fold into a hairpin. Secondary structure analyses showed that the hairpin is a dynamic structure with multiple functional shapes formed by stems and loops, contains sites under positive selection and covariable sites. Accumulation and tolerance of mutations in the intergenic region is a general feature of orthotospoviruses and might mediate adaptation to host plants and insect vectors.

## 1. Introduction

Orthotospoviruses (genus *Orthotospovirus*) are the plant-infecting members of the family *Tospoviridae* (order *Bunyavirales*) [1], their single strand RNA genome is tripartite of negative or ambisense polarity [2,3]. Plants infected by orthotospoviruses suffer severe stunting with marked reductions in yield and quality [4,5,6].

Orthotospoviruses have broad host range, infecting more than 1090 plant species over 90 families [7] that include several important vegetables, legumes, ornamental crops, and weeds. Tomato spotted wilt virus (TSWV) is particularly remarkable. Among orthotospoviruses, TSWV has a worldwide distribution, and the widest host range consisting of more than one thousand plant species, including both monocots and dicots, in over 95 families [6,8]. Orthotospoviruses are transmitted in a circulative-propagative manner [4,9,10] by insects in the order *Thysanoptera* (thrips), mainly belonging to the genera *Frankliniella* and *Thrips* [4,10,11]. There are more than 5000 species of thrips. However, only species in 10 genera are orthotospovirus vectors [6]. Nine species are vectors to TSWV although some isolates are preferentially adapted to local populations of thrips [12]. In contrast, several orthotospoviruses are transmitted by a single vector species [4]. This contrast indicates adaptation of orthotospoviruses to vector transmission.

Viruses have efficient genome architecture optimized for vector transcription, translation, replication, and spread, which includes polycistronic mRNAs [13], overlapping open reading frames [14,15], formation of polyproteins, regulatory RNA structures in coding and non-coding regions [16]. The genome of orthotospoviruses is organized in three single-strand RNAs of negative of ambisense polarity. Based on their size, genomic RNAs are named large (L), medium (M), and small (S). On average their size is 8.8 kb for large (L), 4.8 kb, and 2.9 kb for medium (M) and small (S) RNA segments, respectively [17]. Orthotospoviruses form spherical, membrane bound particles (80–120 nm in diameter) that contain all three genomic RNA segments [5]. 

The orthotospoviral genome encodes four structural proteins and two non-structural proteins [16]. The L RNA is of negative polarity and, after transcription, is translated into the RNA-dependent RNA polymerase. Segment M is ambisense and encodes the precursor of two glycoproteins (Gn and Gc), and non-structural protein M (NSm) involved in cell-to-cell movement in plants [18]. Segment S is ambisense and encodes the nucleocapsid (N) protein and non-structural protein S (NSs), which is a suppressor of gene silencing [19]. Segments M and S contain two non-overlapping open reading frames in opposite polarities that separated by an intergenic region (253–620 nt long) that is highly rich in A and U stretches, and folds into a stable hairpin structure [20]. These hairpins serve as transcription termination signals and 3′ UTR elements that regulate translation [21].

The interaction between orthotospoviruses and their host plants and insect vectors might contribute to the emergence of new strains and possibly to the emergence of new species [12,22]. Virus adaptations to replicate in a host plant might result in a fitness cost in other host species and heterogeneous environmental conditions [23,24,25,26,27]. Because orthotospoviruses replicate in their vector [9], mutations that favor transmission by one species might compromise transmission efficiency in other species [12]. Multiple host plants, in combination with multiple insect vectors, create heterogeneous selection pressure in virus populations [12,22,28]. Despite multiple and heterogeneous selection constrains, orthotospoviruses maintain a wide range of host plants and insect vectors, and new species continue to emerge [22,29,30,31], suggesting that the orthotospovirus genome is mutationally robust and has a remarkable way to adapt to new host plants and insect vectors. However, the genetic factors that mediate adaptation to a diverse array of hosts and vectors remains poorly understood, and the genome-wide variation in orthotospoviruses has not been characterized.

Characterization of genetic variation is fundamental to our understanding of virus evolution and host adaptation [32,33]. Here we profiled genomic variation in all orthotospoviruses using single nucleotide variation, nucleotide diversity, and selection analyses (Supplementary Figure S1). Results showed that the genome of orthotospoviruses contains hypervariable areas at homologous locations across species. The highest variation mapped to the intergenic region in genomic RNA segments S and M. The hairpin formed by the intergenic region of segment S contains areas under positive selection and covariant sites that mediate the formation of multiple structures.

Positive selection and flexibility in the hairpin topological structures is consistent with evolutionary constraints imposed by diverse host plants and insect vectors. Structural flexibility might provide mutational robustness and allow conservation of biological functions. Results presented here show that the intergenic region in RNA segments S and M accumulate and tolerate mutations and might be a major determinant of host plant and insect vector adaptation in orthotospoviruses.

## 2. Results

### 2.1. Orthotospovirus Phylogeny

**Figure 1:**
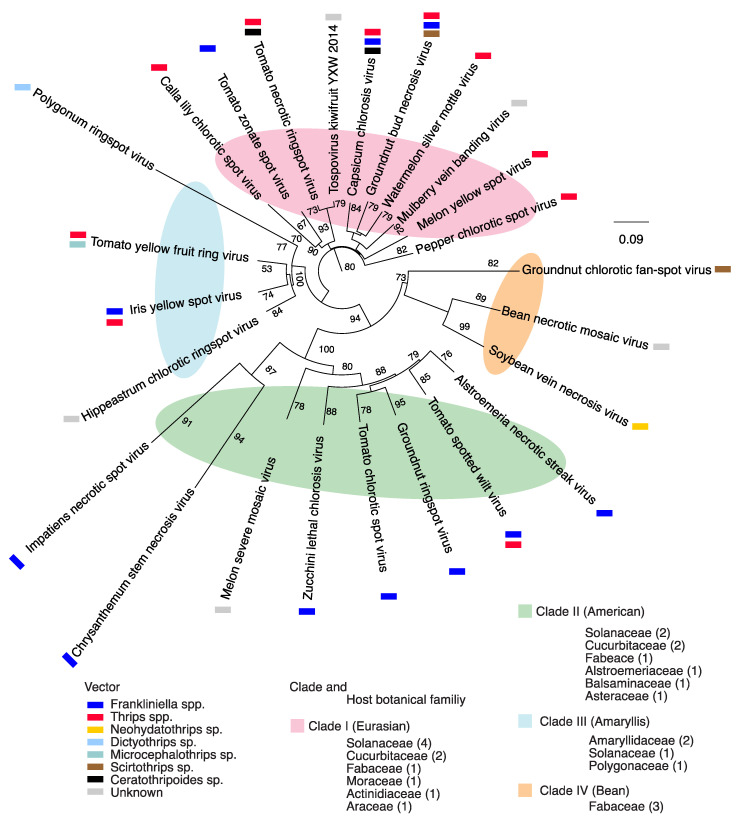
Phylogeny of the genus Orthotospovirus based on segment L and generated by neighbor-joining bootstrap using consensus nucleotide sequences. Colored ellipses indicate clades, which are correlate with the vector, botanical family of the hosts, and geographical origin. The substitutions per sequence sites observed, was 0.09 after 100 bootstraps. In each botanical family the number of viruses is indicated for each cluster. Scale bar represents nucleotide substitutions per site. Vectors are based on Oliver and Whitfield, 2016.

To determine the relationship across species, separate phylogenetic analyses were completed based on segments L, M, or S. A phylogeny based on segment L grouped 25 orthotospoviruses into four clades that correlate with the vector species, geographical origin and the botanical family of the host plants (Figure 1). Clade I included species mainly transmitted by thrips in the genus *Frankliniella* [4], infect plants in the families *Solanaceae*, *Cucurbitaceae*, and *Fabaceae*, and include capsicum chlorosis virus (CaCV), groundnut bud necrosis virus (GBNV), pepper chlorotic spot virus (PCSV), and melon yellow spot virus (MYSV). Previously, species in this clade formed a Eurasian clade based on concatenated amino acid sequence of the RNA-dependent RNA polymerase (RdRp), protein N, non-structural protein S (NSs), and the glycoprotein [34]. Clade II was formed by species mainly transmitted by vectors in the genus *Thrips* [4], and that infect plants in the families *Solanaceae, Cucurbitaceae, Fabaceae*, and include TSWV, impatiens necrotic spot virus (INSV), groundnut ringspot virus (GRSV), tomato chlorotic spot virus (TCSV). These species previously formed an American clade [34]. Viruses in Clade III are transmitted by vectors in the genera *Frankliniella*, *Thrips*, or *Microcephalothrips* [4]: Polygonum ringspot virus (PRV), hippeastrum chlorotic ringspot virus (HCRV), iris yellow spot virus, and tomato yellow fruit ring virus. Viruses in Clade IV are transmitted by vectors in the genera *Scirtothrips* or *Neohydatothrips* [4]: Soybean vein necrosis virus (SVNV), bean necrotic mosaic virus (BNMV) and groundnut chlorotic fan-spot virus (GCFV). Clades III and IV include monophyletic lineages described before [34,35].

A phylogeny based on segment M grouped 28 orthotospoviruses into four clades that correlate with the vector’s species, geographical origin and the botanical family of the host plants (Figure S2), and that largely overlap the clades formed using segment L (Figure 1). A phylogeny based on segment S, grouped 27 orthotospoviruses into three clades (Figure S3) that partially overlap the clades formed using segment L (Figure 1). Members of segment L clade IV grouped together at one end of clade II.

All three-phylogenetic trees obtained from full length nucleotide sequences (Figure 1 and Figures S2 and S3) suggest a polyphyletic topology [36] where viruses within a group do not have common ancestry in all genomic segments. This is consistent with the possibility of multiple ancestors participating through RNA recombination and reassortment [37]. Interestingly, the vector species was an important contributor to the phylogenetic organization (Figure 1), suggesting that there is adaptation for efficient vector transmission. Additionally, difference in tree topology across each genomic segment (Figure 1 and Figures S2 and S3) suggests different evolutionary constraints on each genomic RNA segment. Consistent with this observation, after 100 bootstraps, the nucleotide substitution rate was 0.09, 0.08, and 0.2 for segments L, M and S respectively (Figure 1 and Figures S2 and S3), suggesting that segment S has the highest nucleotide variation rate, and the highest mutational robustness.

### 2.2. Segment S Is the Most Variable

**Figure 2:**
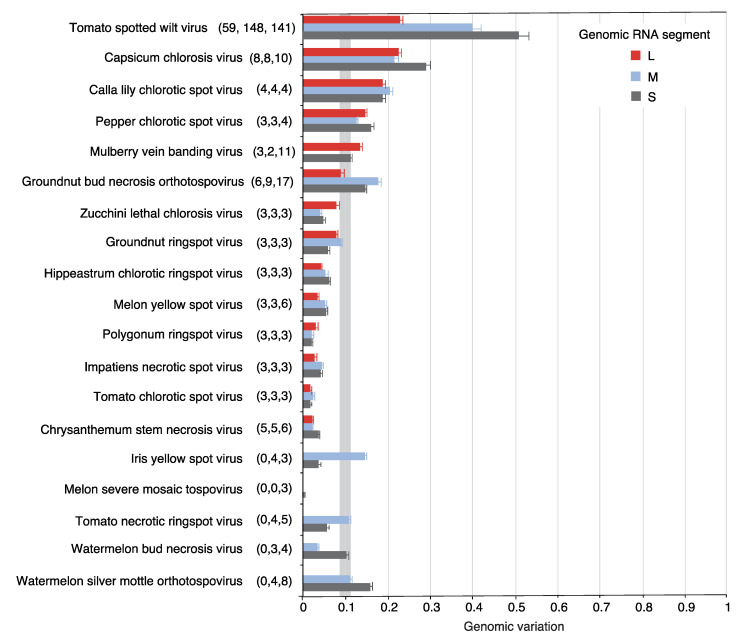
Genomic variation in orthotospoviral RNA. Nucleotide variants were measured separately for segments L, M, and S. Bars represent the genomic variation index, expressed as the proportion of polymorphic sites relative to the length of the segment. For each species, the number of nucleotide accessions for each segment (L, M, and S) are indicated in parenthesis. The gray vertical line represents the mean and a 99% confidence interval (*p*-value < 0.01).

Nucleotide variation in the orthotospoviral genome was determined using genomic variation index, single nucleotide polymorphism, and nucleotide diversity (Pi) analyses [32]. At least three accessions for all genomic segments were obtained for 19 orthotospoviruses. In 12 species, the genomic variation index was at least 10% of the genome (Figure 2). TSWV displayed the highest variation. Interestingly, segment S showed higher variation (50%) than segments M (40%) and L (22%). A similar pattern was observed in seven other viruses. Viruses that deviated from this pattern had a small number of accessions and their genomic variation was less than the observed for the entire genus *Orthotospovirus* (Figure 2).

**Figure 3:**
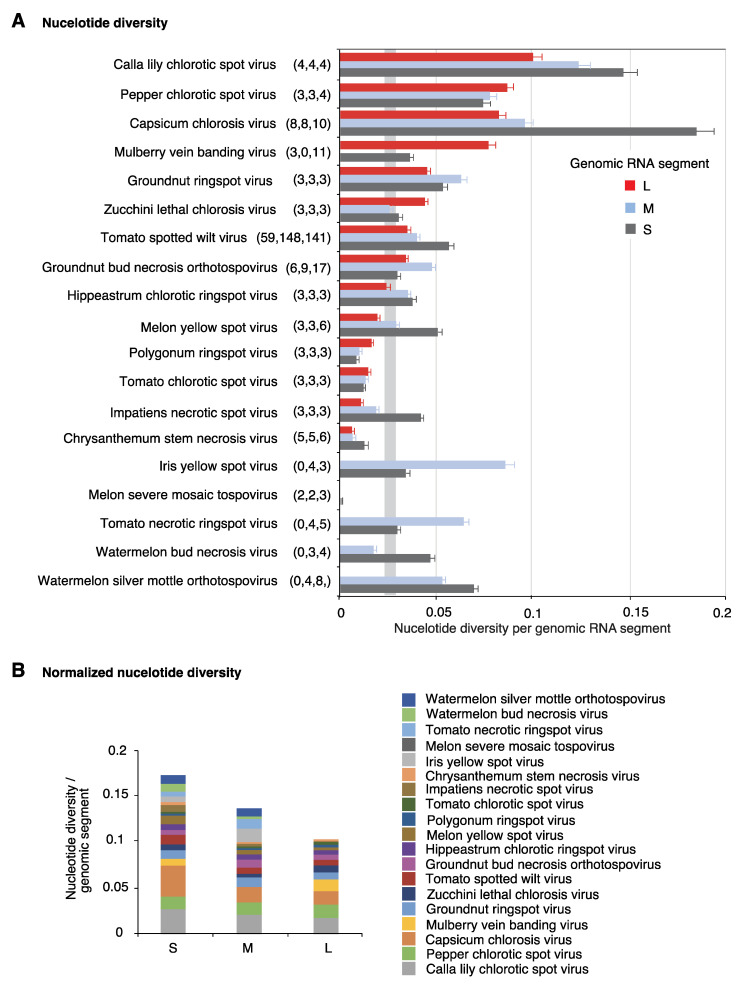
Nucleotide diversity (Pi) in orthotospoviruses. (**A**) Pi was measured separately for each genomic RNA segment (L, M and S). Bars represent the proportion of variable positions with respect to the length of the genomic segment normalized to the number of accessions. For each species, the number of nucleotide accessions is indicated in parenthesis. A confidence interval (*p*-value < 0.01) is plotted as a vertical gray line. (**B**) Cumulative nucleotide diversity normalized to the length of the genomic RNA segment.

The genomic variation index follows a saturation curve [32]. Thus, the unequal nucleotide variation within genomic RNA segments of the same species might be due to unequal number of accessions available for the analysis. To remove the effect of the number of accessions, we estimated variation using nucleotide diversity (Pi), a parameter that corrects for the number of accessions [38]. Results showed uneven variation both across virus species, and across genomic RNA segments of the same virus species (Figure 3A). Segment S harbored higher variation than segments M and L in nine virus species. Variation in segment M was higher in four species, and variation in segment L was higher only in three species (Figure 3A). Genomic segments L, M, and S vary in length within and across species. Thus, we normalized nucleotide diversity to the length of each genomic segment, for each virus species. Values were accumulated for all species analyzed. Result showed that genomic segment S is more variable than M and L (Figure 3B).

Collectively, nucleotide variation estimated through genomic variation index (Figure 2), nucleotide diversity per species (Figure 3A) and by genomic segment (Figure 3B) showed that in orthotospoviruses segment S is the most variable, followed by segments M and L.

### 2.3. Positive and Negative Selection on the Orthotospoviral Genome

The RNA-dependent RNA polymerase (RdRp), NSm, NSs, N, and glycoproteins have specialized roles [4] and interact with host factors [18] that are likely genetically diverse across host plants. These observations predict that cistrons coding for each protein in the orthotospoviral genome are under different selection pressure. To characterize the selection pressure in all cistrons, the frequency of codons under positive or negative selection was determined for the top ten orthotospovirus species with the most variation. Sites under positive or negative selection were estimated for each cistron, and frequencies normalized to the length of the open reading frame.

**Figure 4:**
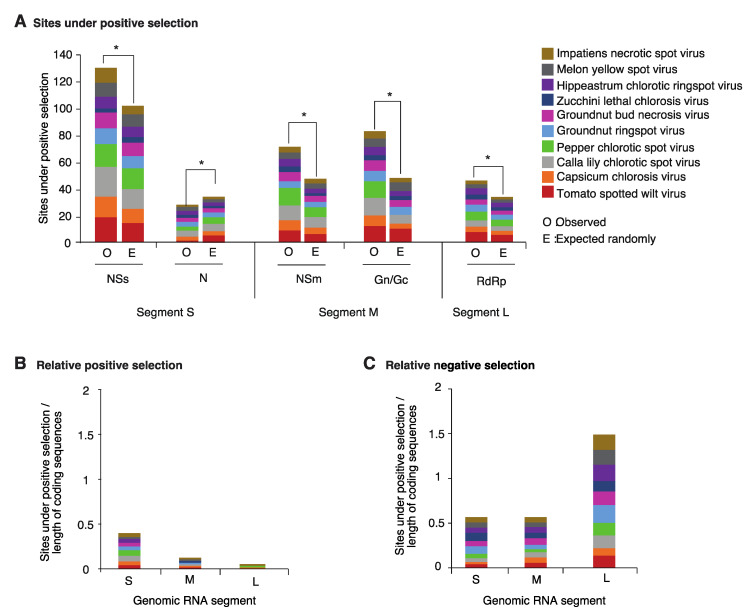
Positive and negative selection in the top ten most variable orthotospoviruses. (**A**) Frequency of the sites under positive selection normalized to the length of the cistron compared to the expected randomly (sites per cistron / total for the open reading frame). * denotes significant differences with *p*-value ≤ 0.001 as calculated by the Chi-square test. (**B**) Relative number of sites under positive selection for each species, expressed cumulatively by genomic RNA segment, and normalized to the length of the open reading frame. (**C**) Relative number of sites under negative selection.

NSs is the cistron with the highest number of sites under positive selection. The frequency is higher than the expected randomly (Figure 4A). Cistrons coding for NSm, the glycoprotein, and the RdRp accumulated sites under positive selection at a rate that is higher than expected randomly. However, the RdRp cistron was less variable that NSs, the glycoprotein and NSm. In contrast, the cistron coding for nucleocapsid protein N was the most stable and accumulated sites under positive selection at a rate that is below the expected randomly (Figure 4A).

The number of sites under positive or negative selection were normalized to the length of the open reading frame and plotted by genomic segment for the top ten orthotospovirus species with the most variation. Codons under positive selection in RNA segment S are 3 and 6 times more abundant than in segments M and L, respectively (Figure 4B). In contrast, the proportion of codons under negative selection in RNA segment L is 3 times higher than the observed for segments S and M (Figure 4C). Thus, based on positive and negative selection analyses, genomic RNA segment L is the most genetically stable, and segment S is the most variable.

### 2.4. Nucleotide Variation in Segment S

**Figure 5:**
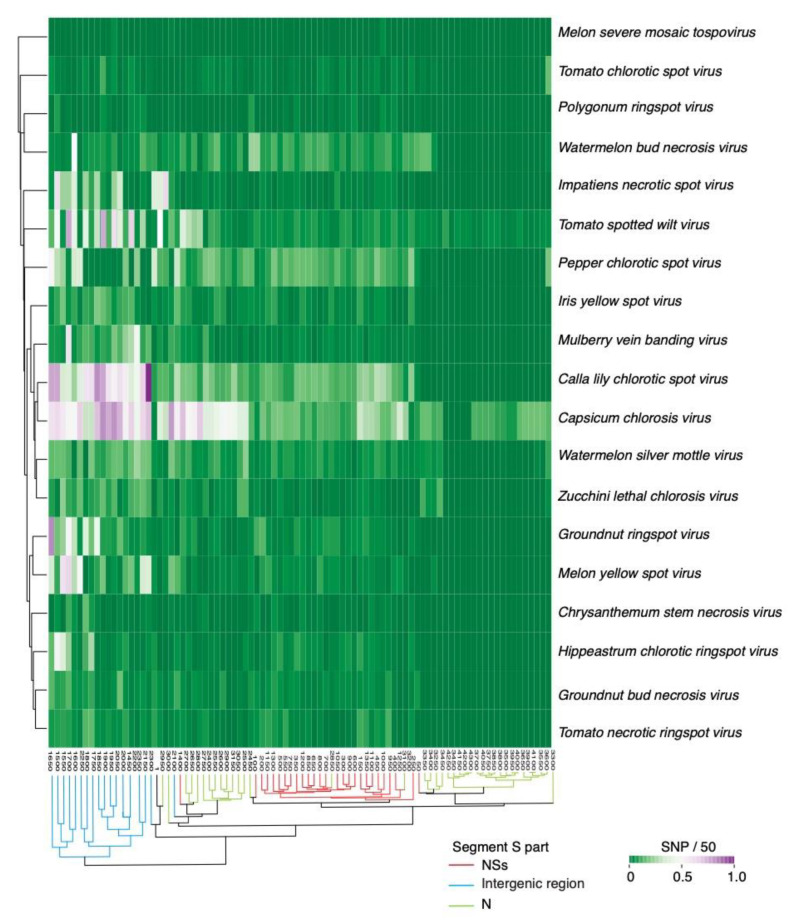
Two-way hierarchical clustering based on single nucleotide polymorphism in genomic RNA segment S from orthotospoviruses. The Y-axis correspond to virus species. The X-axis represents coordinates. Clusters separate the NSs cistron, intergenic region and nucleocapsid cistron.

Mutations could be distributed randomly or localized to form hypervariable areas. To distinguish the difference, we performed a two-way cluster analysis using segment S from all orthotospoviruses and single nucleotide polymorphisms were identified in a 50-nt window. Across orthotospoviruses, the intergenic region accumulates the highest frequency of mutations (Figure 5). Accordingly, hyper variation in the intergenic region is a general feature of orthotospoviruses.

Clades formed by a segment S phylogeny (Figure S3) did not match the groups formed by the two-way variation analysis (Figure 5). This difference suggests that the distribution of mutations in the intergenic region and in the two cistrons of segment S is independent from taxonomic relationship between orthotospovirus species.

### 2.5. Genome-Wide Variation in Tomato Spotted Wilt Virus

TSWV has the widest host range amongst both orthotospoviruses and in all plant viruses [6,8]. To identify and characterize the distribution of mutations in the TSWV genome, single nucleotide variation and nucleotide diversity were estimated on a 50-nt window and mapped to each genomic segment. Additionally, for the cistrons in each segment, positive selection analyses were performed using SLAC and MEME. Codon sites predicted by both methods were considered under selection [32].

**Figure 6:**
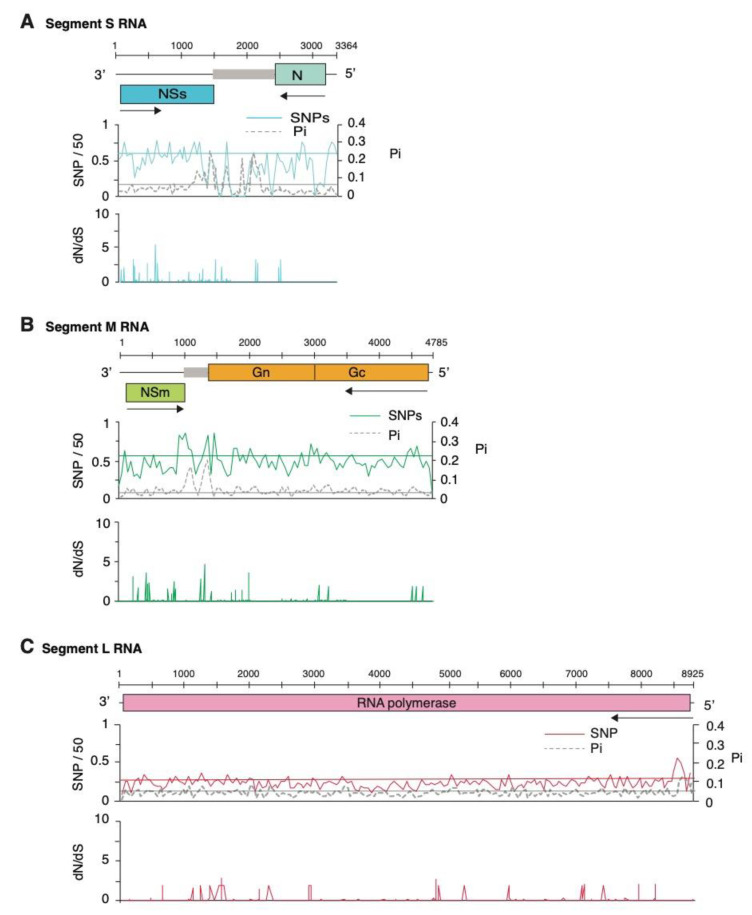
Genome-wide variation in Tomato spotted wilt virus. Single nucleotide polymorphism (SNP) and nucleotide diversity (Pi), and the ratio of non-synonymous to synonymous changes (dN/dS) were estimated in 50-nt window. The average and a 99% confidence interval (*p*-value < 0.01) is indicated as a horizontal line. Gray squares in the intergenic region of S and M indicate transcription termination hairpins. (**A**) Genomic RNA segment S. Coordinates are based on accession AJ418778.1. (**B**) Genomic RNA segment M. Coordinates are based on accession KT717692.1. (**C**) Genomic RNA segment L. Coordinates are based on accession MF159042.1.

In TSWV, nucleotide variation is not equally distributed between genomic RNA segments and is not randomly distributed within each segment (Figure 6). In segment S, both nucleotide variation and nucleotide diversity identified hypervariable areas in the intergenic region, and at the C-terminal part of the NSs cistron (Figure 6A). In contrast, variation in the nucleocapsid cistron, the 5′ UTR and the 3′ UTR were below the average for the genome (Figure 6A).

Genomic RNA segment M is second in variation to segment S (Figure 3B and Figure 4). Distribution of nucleotide variation and nucleotide diversity along segment M showed that variation mainly maps to the intergenic region (Figure 6B), with variation higher than average also detected at the C-terminal of NSm, at the ends of the glycoprotein and near the protease cleavage site in the glycoprotein (Figure 6B). Glycoprotein variation might be related to its interaction with thrips vectors [39,40].

Segment L showed contrastingly lower variation than segments S and M, with only the C terminal end of the RdRp cistron showing signs of variation (Figure 6C). Thus, the RdRp is remarkably genetically stable, consistent with conserved functions during virus replication in host plants and in insect vectors [41]. Selection analysis based on dN/dS (>1, *p*-value ≤ 0.05) displayed the presence of codons under positive selection in NSs and NSm cistrons. A lower number of sites under positive selection were detected in cistrons coding for the glycoprotein and for the RdRp (Figure 6).

Contrasting difference between genomic segments might indicate different sources of selection constraints imposed by vectors, hosts, and possible roles of different parts of the genome in host and vector adaptation. The intergenic region in segments M and S folds into a hairpin that regulates transcription termination translation [20,21]. Remarkably, both in segments M and S the intergenic region is hypervariable (Figure 6B), pointing to this part of the genome as a major determinant of host and vector adaptation.

### 2.6. Genome-Wide Variation in Other Orthotospoviruses

Nucleotide variation and positive selection analyses were mapped for nine orthotospoviruses with highest variation. In CCV RNA segment S, the highest variation mapped to the intergenic region, the C-terminal of the NSs cistron, and the C-terminal part of the N cistron. In the NSs and N cistron, sites under positive selection mapped to hypervariable areas (Figure S4A). Less variation was observed in segment M (Figure S4B). However, the variation concentrated at the intergenic regions, and at the C-terminal part of NSm. Codons under positive selection were less frequent than in segment S and were scattered across NSm and the glycoprotein cistrons (Figure S4B). Segment L showed the lowest variation, and the highest peak was near the stop codon and included the 3′ UTR (Figure S4).

In CLCSV, within segment S, the highest variation mapped to the N and C-terminal parts of NSs and N cistron respectively, and the intergenic region (Figure S5A). Hypervariable regions within RNA segment M mapped to C-terminal part of NSm and intergenic region, with less variation observed at cistron coding for the glycoprotein (Figure S5B). L segment showed a high peak near the stop codon and included the 3′ UTR (Figure S5C). Genome-wide distribution of variation followed a similar pattern in PCSV, GBNV, ZLCV, GRSV, HCRV, MYSV, and INSV (Supplementary Figures S6–S12).

Collectively, genome-wide distribution of nucleotide variation, estimated by three different methods, showed that orthotospovirus contain hypervariable regions at homologous locations in the genome, with the highest variation mapping to the intergenic region of segments S and M (Figure 6 and Figures S4–S12).

### 2.7. TSWV Genetic Diversity

TSWV is the most widely distributed orthotospovirus and has the widest range of host plants and insect vectors [6,8]. In TSWV, as in orthotospoviruses in general, genomic segments S and M are the most variable (Figure 3 and Figure 6). To determine if variation in genomic segments S and M correlate with host or geographical location in TSWV, a phylogeny was obtained based on full length RNA sequences for segments S and M. For genomic RNA M, accessions from Eurasia and the America formed separate clusters. However, no clusters were formed based on host plant or country (Figure S13A). For genomic RNA S, accessions from Eurasia and the America formed distinct clades. Accessions from the same host formed clusters, and accessions from the same country or region within a country generally clustered together (Figure S13B). Accordingly, variation within segment S might be correlated to host plants, insect vectors, or their combination.

**Figure 7:**
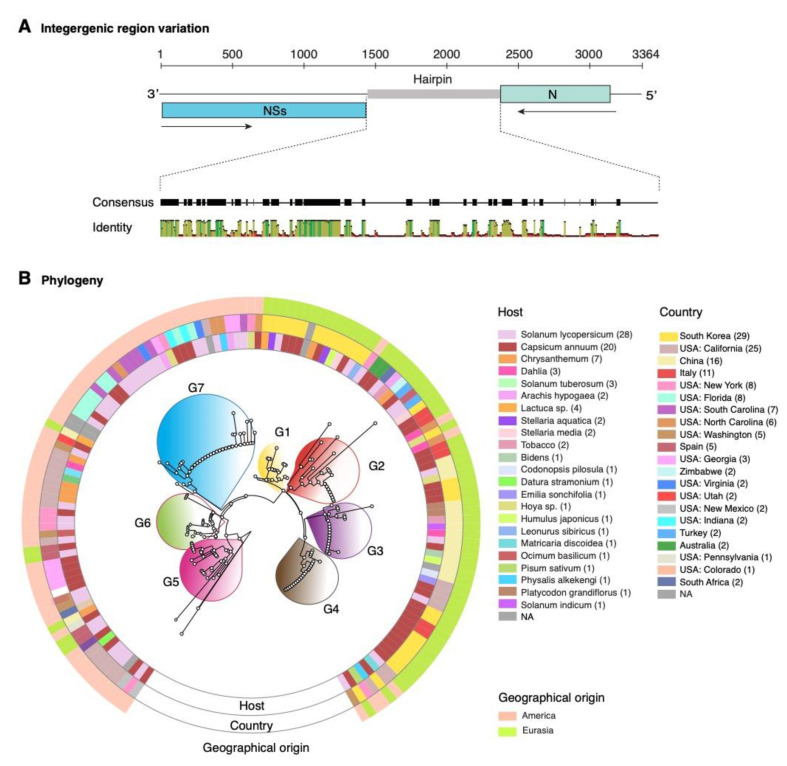
Nucleotide sequence variation in TSWV RNA segment S intergenic region. The analysis was based on 141 accessions aligned using LocRNA and the phylogenetic tree was generated using MAFFT. (**A**) Graphical representation of segment S. Coordinates are based on accession AJ418778.1. Consensus and identity plots for the intergenic region were generated with LocRNA were visualized in Geneious. (**B**) Phylogram based on intergenic region nucleotide sequences. Groups formed are numbered and color-coded in the center. Within each group, sequence similarity is more than 95% (*p*-value < 0.01). Outer rings indicate host, country of origin and geographical clade.

To further characterize variation in TSWV segment S, for all accessions available, we aligned the sequences corresponding to the intergenic region. High polymorphism was detected and included insertions, deletions and nucleotide substitutions (Figure 7A). Consistent with the phylogeny based on the entire segment S (Figure S13), two distinct groups of TSWV isolates were identified that correlated (match cutoff probability *p* = 0.01) [42] with geographical origin (Eurasian or American, Figure 7B).

Eurasian isolates formed four groups. Group 1 contained accessions from South Korea infecting several host plants. Group 2 was the most diverse and included accessions from eight countries and collected from pepper, tomato, tobacco, or chrysanthemum. Group 3 included mainly accessions from China and three from South Korea collected from several hosts. Group 4 included mainly accession from South Korea, and some from Italy or the USA collected mainly from pepper, physalis, pea, and tomato (Figure 7B).

American isolates formed three groups, numbered 5 through 7. Group 5 contained accessions mainly from California and one from Georgia, collected from tomato, pepper, or datura. Group 6 contained accessions mainly from Georgia, New York, and one from Washington, collected from tomato, pepper, or chrysanthemum. Group 7 contained the largest number of accessions, mainly from Florida, South Carolina, Virginia, and Indiana, collected from tomato, pepper, peanut, or physalis (Figure 7B).

These observations suggest that mutations in the intergenic region of genomic segment S in TSWV support structural diversity in the hairpin and might reflect selection pressure to adapt to genetically diverse host plants, insect vectors, environmental conditions, and their combinations.

### 2.8. Nucleotide Variation in Segment S Intergenic Region

**Figure 8:**
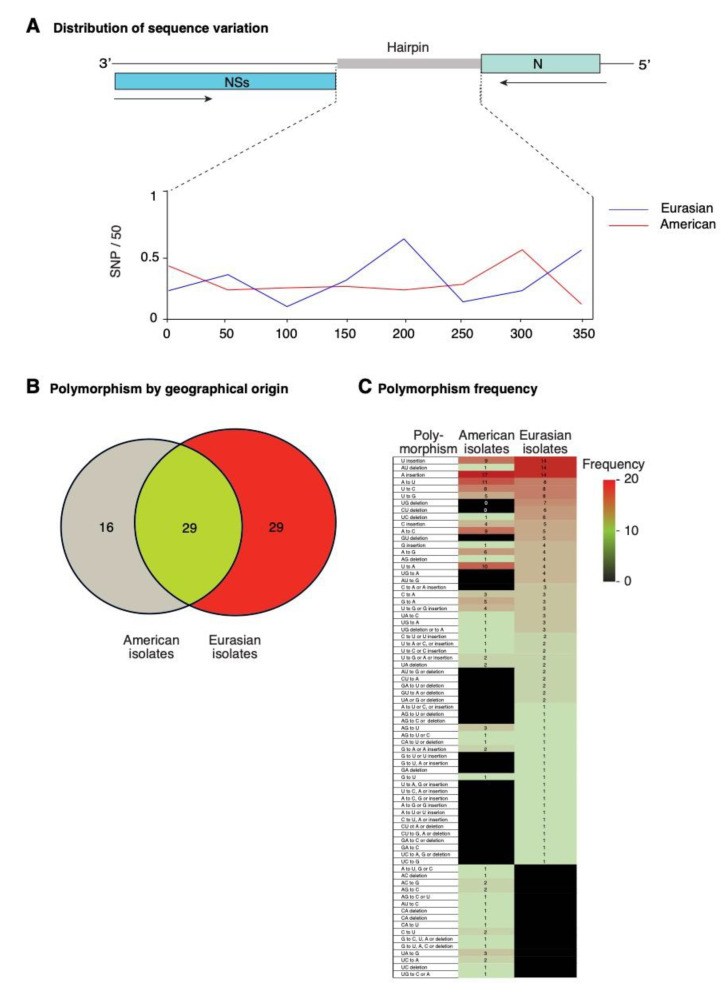
Distribution and characterization of polymorphisms in the intergenic region of TSWV segment S. Accessions (n = 141) were separated by geographical origin into Eurasian and American clusters. Polymorphisms include nt substitutions, insertions, and deletions. (**A**) Distribution of single nucleotide polymorphism estimated in a 50-nt window. (**B**) Number of common and clade-specific polymorphisms. (**C**) Kind and frequency of polymorphisms with respect to the reference (NC_002050.1) and by geographical group.

To characterize the differences between TSWV isolates of Eurasian and American origin, we performed a single nucleotide polymorphism analysis focused on the intergenic region of segment S (Figure 8). Both frequency and distribution of mutations are different between Eurasian and American isolates. A total of 293 and 144 polymorphic sites were identified for Eurasian and American isolates, respectively. In Eurasian isolates, the highest frequency of nucleotide variation mapped to the middle if the intergenic region. In contrast, in American isolates the highest frequency of nucleotide variation mapped to areas near the 3′ and 5′ ends of the intergenic region (Figure 8A).

### 2.9. Structural Flexibility in Segment S Hairpin

The diversity in nucleotide sequence (Figure 7) suggests that there is diversity in hairpin structures. To test this hypothesis, we compared nucleotide sequence and predicted structure for all TSWV accessions available. The process considers an energy model for RNA secondary structure using Zuker’s fold algorithm [42].

For each group formed using segment S complete nucleotide sequence (Figure 7B), the consensus sequence of the intergenic region was obtained. Secondary structural modeling showed that the segment S intergenic region folds into a hairpin of diverse topological structures that contain co-variant sites (Figures S14 and S15). The most common structure was a rod-like hairpin. Branched Y structures were less common (Figures S14 and S15).

**Figure 9:**
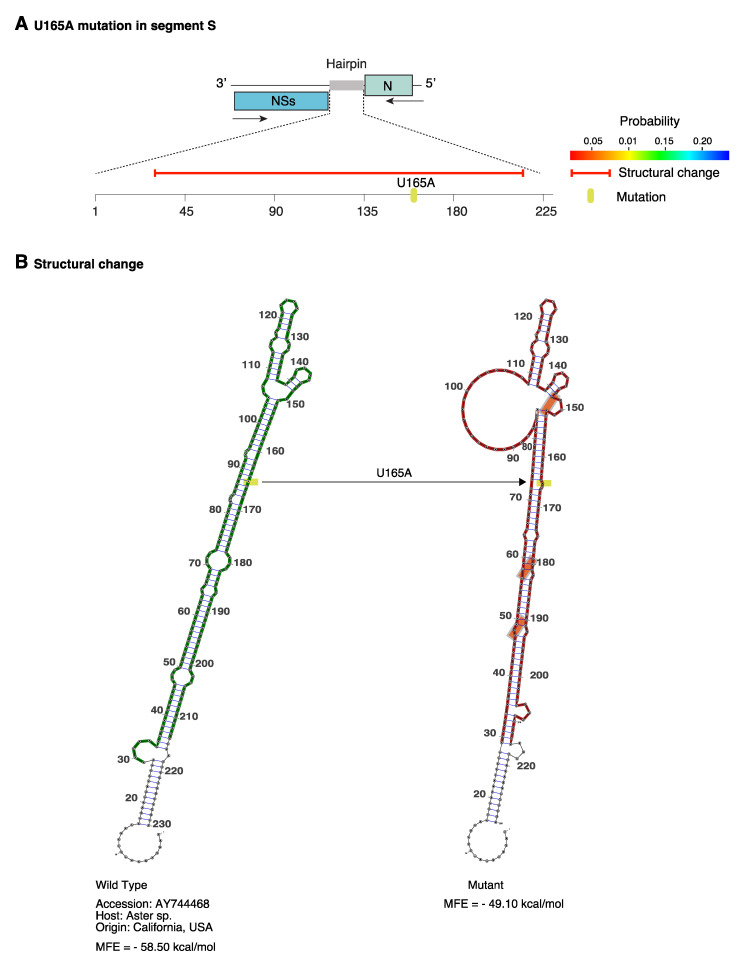
Representation of the structural changes in TSWV segment S hairpin caused by the U165A mutation. Using RNAmute, the model was generated for accession AY744468 and naturally occurring mutation (U165A). (**A**) Representation of the hairpin, the mutation, and the predicted effect on the secondary structure. The red horizontal line represents predicted structural change. The probability of the structure is color coded. (**B**) Hairpin structure in wild type and mutant sequence. The U165A mutation is indicated in yellow. Orange boxes indicate covariant sites predicted to emerged after the initial mutation.

In an alternative approach, we modeled structures in the hairpin of accession AY744486. Mutation U165A was frequent in American isolates (Figure 8C). The model in RNAmute [43] predicted that a single U165A mutation caused structural changes in 80% of the hairpin (Figure 9A), resulting in a new topology. The model also predicted that covariant sites provide stability to the new structure (Figure 9B). This result supports the hypothesis that segment S hairpin might exist as multiple functional structures across TSWV isolates.

Mutations were classified into nucleotide insertions, deletions, or substitutions. An overlap analysis identified 58 and 45 and kinds of polymorphisms in Eurasian and American isolates, respectively. Of those, 29 were common, 29 were exclusive to Eurasian isolates, and 16 were exclusive to American isolates (Figure 8B). The most frequent mutations were single nucleotide insertions or deletions. A to U, and U to G transversions, and U to C transition were the most abundant nucleotide substitution detected both in American and Eurasian isolates. The U to A transversions was amongst the most frequent in American isolates, and less frequent in Eurasian isolates. Two-nucleotide deletions occurred at higher frequency in American isolates than in Eurasian isolates. Substitutions exclusive to a particular group of isolates occurred at low frequency (Figure 8C).

To further asses structural diversity in TSWV segment S hairpin, models were generated for several accessions from Eurasia and America using two independent algorithms: RNAmute [43] and RNAsnp [44]. These algorithms are able to detect the deleterious polymorphisms as well as the positions on RNA prone for structural changes [43,44].

**Figure 10:**
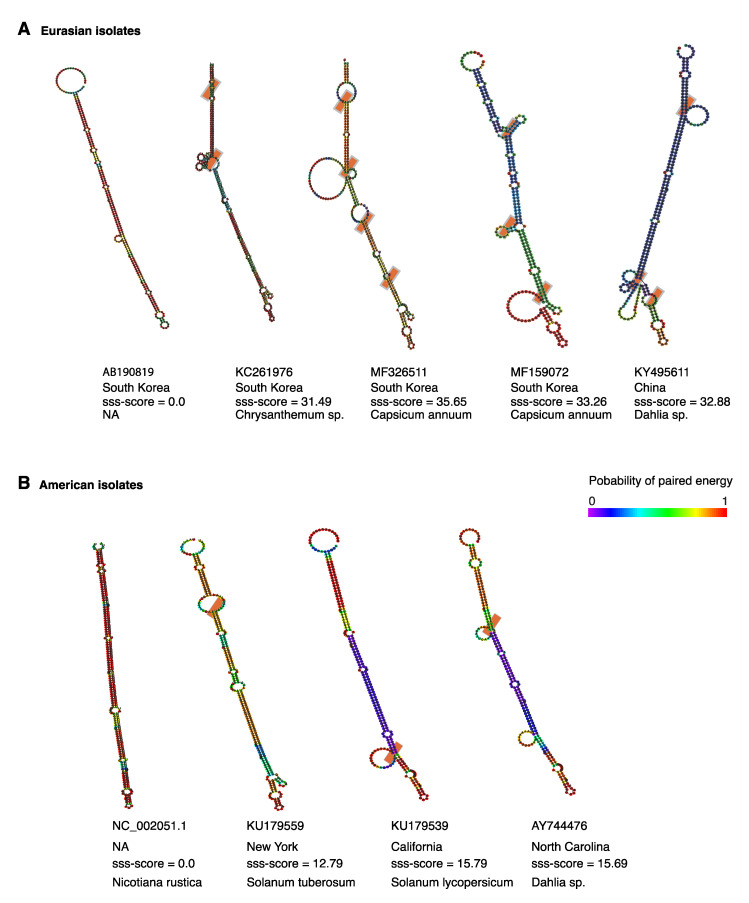
Representative models of TSWV segment S hairpin in Eurasian and American isolates. Structures were modeled using SSS-test to asses positive and negative selection on RNA secondary structure. Low and high sss-scores indicate negative and positive selection, respectively. Colors indicate probability of paired energy. Orange boxes indicate covariant sites. (**A**) Representative Eurasian isolates. (**B**) Representative American isolates.

The hairpin folded into diverse topological structures that contain co-variant sites. The most common structure was a rod-like hairpin (Figure 10).

Collectively, these results showed that there is diversity in segment S hairpin RNA sequence and secondary structure. Consistent with these results, structural flexibility has been observed both in the segment M and S hairpin in TSWV [45].

### 2.10. Positive Selection at Intergenic RNA Structures in TSWV

Selection pressure might favor mutations that support the formation of functional secondary structures [46]. It is possible that the hairpin in segments S and M of orthotospoviruses is under positive selection. We tested this hypothesis using SSS-t [46] to quantify positive and negative selection on the TSWV segment S hairpin (Figure 10). Using a stringent selection score cutoff of s ≥ 10.0, 46 out of 69 (66.6%) local structures showed signs of positive selection in Eurasian isolates (Figure 10A). High positive selection scores with low paired energy were detected for accessions from South Korea, and China with an excess of structure changing substitutions leading to structural variations (Figure 10A). In isolates of American origin, flexible structures under positive selection were detected in 21 of 71 isolates (29.6%). The positive selection scores were lower, compared to the observed from Eurasian isolates (Figure 10B).

These results indicate that the hairpin formed by the intergenic region in TSWV segment S is structurally flexible and is under positive diversifying selection. Selection pressure is higher in Eurasian isolates than in American isolates (Figure 10).

## 3. Discussion

Sources of variation in RNA viruses are linked to virus replication an include nucleotide insertions, deletions, and substitutions introduced by the viral RNA-dependent RNA polymerase during RNA synthesis [47]. RNA recombination also contributes to the generation of genetic variation and occurs during replication [48]. Additionally, in viruses with a segmented genome, such as orthotospoviruses, reassortment of genomic RNA segments contributes to the generation of genetic diversity [37,49]. Genetic variation generated through these mechanisms constantly creates new variants that are the raw material for selection [50]. In viruses, host adaptation is an evolutionary process linked to the balance between genetic variation and selection. The outcome is the emergence of new viral strains or species [22,33,50,51,52].

Mutations introduced by the viral RNA-dependent RNA polymerase during replication might occur randomly. However, the phenotypic effect of those mutations on fitness is not random [32,33]. Due to their neutral effect on fitness, synonymous substitutions are likely to be maintained. In contrast, purifying selection acts on mutations that affect fitness. While non-synonymous mutations causing deleterious effects are removed from the population, mutations that provide an adaptive advantage are favored and their frequency in the population increases [53,54]. Because diverse hosts and vectors impose heterogeneous selection constraints on viral genomes [26,28,54], viral genes that mediate host adaptation are hypervariable [32,33]. Consistent with this model, in several plant and animal viruses, factors that determine virulence, and suppression of host defenses, are genetically variable and contain sites under positive selection [32,33,51,55]. Furthermore, in several plant viruses, vector transmission efficiency is affected by mutations in viral proteins [12,54,56] Accordingly, after selection, the distribution of mutations in the genome is not random [32,33] and might reflect the footprints of selection.

**Figure 11:**
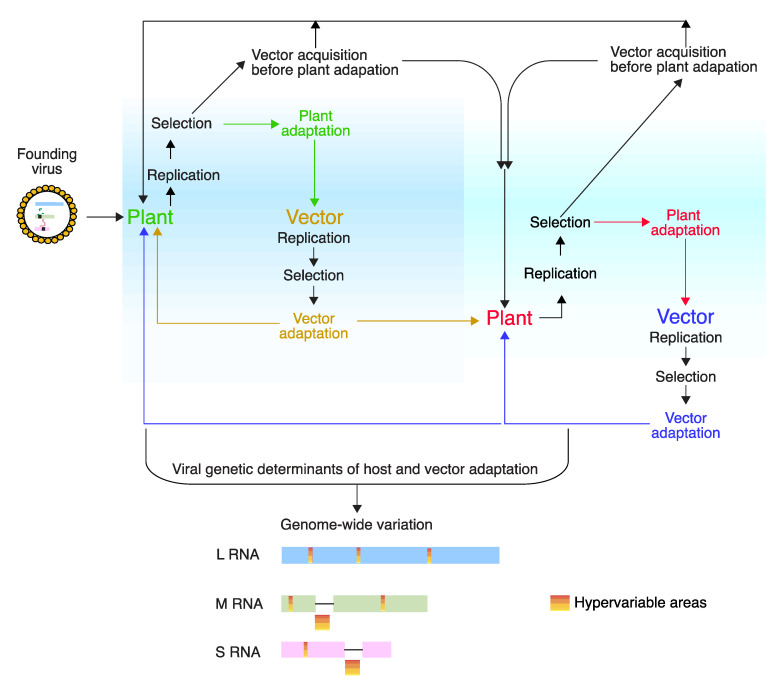
Model for orthotospovirus genomic variation, selection, host and vector adaptation. Replication and movement in plants, replication efficiency in the vector, and transmission efficiency are selection constrains. The vector may acquire the virus before or after adaption to a host plant. Before adaptation, the virus population is more diverse that after host plant adaptation. Variants that replicate and are transmitted efficiently are more likely to infect a new plant of the same or different genotype, in the same or different ecological niche. Alternate cycles of replication and selection between vector and host plant. The diversity of plant and vectors imposes selection for rapid adaptability. Mutations preferentially accumulate in viral determinants of host and vector adaptation. The foot prints of selection can be detected by genome-wide characterization of genetic variation.

The evolutionary arms race between hosts and pathogens results in selection for rapid changes in key genes [57]. Virus adaptations to replicate in a host plant might result in a fitness penalty in other host species or in different environmental conditions [23,24,51,58,59]. Alternatively, or in addition, host and/or vector adaptation without compromising fitness might select for viruses that can rapidly accumulate and tolerate mutations is key areas of their genome (Figure 11).

Because orthotospoviruses replicate in their vector [9], mutations that favor transmission by one vector species might compromise transmission efficiency in other vector species [12,60,61]. Under this scenario, orthotospoviruses are forced to maintain functionality in a diverse and alternate array of plant hosts and insect vectors (Figure 11). Despite these constrains, orthotospoviruses have a wide host range and vector range, and new strains and species emerge continuously [22,29,30,31,62].

Characterization of genetic variation in viruses is fundamental to our understanding of virus evolution and host adaptation [32,33]. The mechanisms of host adaptation in orthotospoviruses are poorly understood, and their genome-wide variation has not been characterized. In this study, we profiled variation in the genome of all orthotospoviruses represented by three or more complete genome accessions. The genome of orthotospoviruses is highly variable. Segments S and L are the most, and the least variable, respectively (Figure 2 and Figure 3). Accordingly, segment L might be useful to determine phylogenetic relationships between orthotospovirus species (Figure 1), while segment S might be useful to distinguish strains within a particular species (Figure 7B).

Within coding regions, the cistron coding for nucleoprotein N is the most genetically stable (Figure 4A). The frequency of sites under positive selection was lower than expected randomly. In contrast, in all other cistrons, the frequency of sites under positive selection was higher than expected randomly. The cistron coding for NSs is the most variable (Figure 4A). Interestingly, in segments S and M, the intergenic region is more variable than the open reading frames flanking it (Figure 5 and Figure 6). In both segments M and S the intergenic region contains an area that is A/U rich and folds into a hairpin structure [20] that functions as transcription termination signal and regulates translation [21,63,64,65]. In segment S, the hairpin enhances translation in concert with proteins N and NSs, and the A-rich stretches mediate binding of poly(A)-tail-binding protein to promote transcription termination [65]. Our results show that hyper variation in the intergenic region is a general feature of orthotospoviruses (Figure 6, and Figures S4–S12).

Among orthotospoviruses, TSWV has the widest host range [6,8] and is transmitted by several species of thrips [4,12]. Accordingly, TSWV is a generalist’s virus. A key feature of generalist pathogens is their high genetic diversity [66]. Interestingly, genetic diversity in TSWV is not randomly distributed. Instead, genetic diversity preferentially accumulates in the intergenic region of segments S and M (Figure 6). These results points to the intergenic in TSWV, and possibly in all orthotospoviruses, as a determinant of host adaptation.

Several lines of evidence showed that hyper variation in segment S intergenic region supports the formation of diverse secondary structures. Sequence and structural analysis of the intergenic region in TSWV segment S separated isolates by geographical origin into Eurasian and American, and into seven groups (Figure 7B). No correlation with the host was detected. Hairpin structures were generated for consensus sequences in each group. For each TSWV group the hairpin formed a unique topology (Figures S14 and S15). In American isolates, the U165A mutation was amongst the most abundant (Figure 8C). This single mutation is predicted to cause a mayor re-arrangement in the hairpin structure (Figure 9B). Modeling of structures for individual isolates of Eurasian or American origin indicates the formation of diverse structures (Figure 10).

Structural conformations in viral RNA regulate binding to RNA or protein interaction partners, and structures formed by intergenic regions participate in diverse biological functions [67,68,69]. Sequences that fold into similar structures may support similar functions, and mutation within these regions will be tolerated only if they preserve a functional structure [57,70].

The term covariation refers to sites that together provide support to an RNA structure. Covariation, in the form of base-pair insertions/deletions might be necessary to stabilize the hairpin structure [68,71,72]. Covariation is sign of evolutionary selection on RNA structure in response to host adaptation [72,73]. Structural modeling of the hairpin in TSWV segment S indicates that covariant sites provide stability of the hairpin structure (Figure 9B).

In segment S the hairpin regulates translation. This process also requires host factors, such as the poly(A)-tail-binding protein [65]. Host factors that participate in translation of segments S and M are likely to be diverse in host plants of different species or genotypes within the same species. Similarly, in insect vectors, factors that participate in translation of viral RNAs are likely to be diverse. Genetic and structural flexibility in the hairpin might provide mutational robustness and a rapid way to generate genetic diversity and maintain functionality.

Consistent with this model, in multifunctional, structured RNAs, a single sequence must adapt alternative secondary structures to execute several functions. These RNAs have abundant covariant sites and are selected for structure/function conservation [74], and alternative structural conformations and covariation were observed both in the segment M and S hairpin in TSWV [45].

During the course of selection and adaptation process, plants select for variants with efficient replication, cell-to-cell movement and systemic movement. Vectors likely acquire a quasispecies [39] and selection is expected to favor variants with efficient replication and transmission efficiency (Figure 11) [75].

Systematic biological experimentation is needed to elucidate the genetic determinants of host plant and insect vector adaptation in orthotospoviruses. Results described here point to the hairpin in the intergenic region of segments S and M, and suggest the hypothesis that the success of TSWV as a generalist pathogen is determined by hypervariable intergenic regions. Consistent with this model, between TSWV isolates from lettuce, pepper, and tomato, the main difference mapped to the intergenic region of segment M [22]. However, cistrons coding for NSs, NSm, the glycoprotein, and the RdRp (Figure 4A) are also likely to be involved. TSWV from lettuce was able to infect maize. During adaptation to maize, in additions to mutation in the intergenic region, mutations accumulated in the Gc part of the glycoprotein encoded by segment M [22]. Furthermore, in the glycoprotein, mutations that inhibit transmission by thrips have been identified [39].

## 4. Materials and Methods

All computational analyses presented in this work (Supplementary Figure S1) was conducted on the high-performance computing nodes at the University of Nebraska-Lincoln Holland Computing Center (https://hcc.unl.edu/). Custom scripts are available upon request.

### 4.1. Genomic RNA Sequences

The available genomic sequences of orthotospoviruses (Supplementary Table S1) were obtained from databases at the NCBI website (http://www.ncbi.nlm.nih.gov/) on September of 2019 using customized scripts based on Entrez Programming Utilities (E-utilities, National Center for Biotechnology Information, Washington, DC, USA; https://eutils.ncbi.nlm.nih.gov/entrez/eutils/). For each species, a random accession describing a complete genome information was used as reference (Supplementary Table S1). Compared to the reference, accessions containing less than 95% of the genome length were considered incomplete and removed. Subsequently, variation analysis was performed only for species with three or more accessions. In-house perl script was developed to make a consensus sequence from all isolates of each species.

### 4.2. Removal of Recombinant Sequences

Putative recombinants were identified using RDP4 (Computational Biology Group, Institute of Infectious Disease and Molecular Medicine, University of Cape Town, Cape Town, South Africa) [76]. Genomic sequence of all orthotospoviruses were analyzed, with a Bonferroni-corrected *p*-value cut off <10^−4^, using GENECONV, 3Seq, SiScan, MaxChi and BootScan, and RDP as implemented in RDP4 [32]. Accessions with a Bonferroni-corrected recombination breakpoint detected at significant *p*-value was discarded before performing next analysis. Additionally, putative reassortment events with RDP4 v.4.80 [76] using several algorithms on the MAFF alignment file of concatenated full-length genome sequences. Sequences identified as reassortants were removed from the dataset.

### 4.3. Complete Genome Sequences and Consensus

A total of 5661 genomic RNA sequences for 38 orthotospoviruses were obtained from NCBI (https://www.ncbi.nlm.nih.gov/) on September of 2019. For statistical relevance [32], only species with three or more complete accessions per genomic segment were considered. For complete segments L, M, and S a total of 107, 216, and 236 number of accessions were included in the analysis. For each virus, one accession with the complete genome was used as reference (Table S1). For each virus species and for each segment, consensus sequences were obtained and used to generate phylogenetic trees by neighbor-joining method [77].

### 4.4. Molecular Phylogeny

The consensus sequence of each genomic RNA (L, M, and S) from each orthotospovirus species was used to construct phylogenetic trees by neighbor-joining method [77] using MAFFT (Bioinformatics Center, Kyoto University, Kyoto, Japan) [78] with 1000 bootstrap replicates.

### 4.5. Polymorphism Analysis in L, M and S Segment

Mafft-derived alignment file of all three genomic sequence from each virus species was used for identification of single nucleotide polymorphism (SNPs) via SNP-sites version 2.4.1 (Pathogen Genomics, Wellcome Trust Sanger Institute, Wellcome Genome Campus, Cambridge, UK) [79]. For each SNP, the details were obtained in a variant call format (VCF). In a 50-nt sliding window, SNP density was obtained using VCFtools [80]. For each genomic segment (L, M, and S) variation index was estimated by normalizing total SNPs to the length of the corresponding genome. In an alternative approach, in nexus format, alignment files for all three genomic segments were used to determine pairwise nucleotide diversity (Pi) in a 50-nt sliding window using the Tajima’s D test available in DnaSP 5.10.1 [38]. For genomic variation and Pi, for each genomic segment, the average and standard error was estimated for each genomic segment. A general average and a 99% confidence interval were estimated to identify species and segments with low and high variation.

### 4.6. Distribution of Variation in Segment S

To determine the distribution of variation in segment S, a two-way cluster analysis was performed. SNPs identified in all orthotospoviruses were used as input for hierarchical clustering using the ClustVis in R. Clusters are generated first by finding the shortest link among all species and coordinates in the genomic segment.

### 4.7. Selection Analysis

For the 14 most variable orthotospoviruses, a selection analysis was performed. The open reading frames of each genomic RNA were aligned with MAFFT. Nucleotide anonymities within the sequences were discarded using a custom bash script. The subsequent alignment file was used to get the rate of non-synonymous and synonymous mutations at each site based on Single-likelihood ancestor counting (SLAC) and MEME using HyPhy [33] with significance level of 0.05 and >0.95 posterior probability. Sites detected by both methods were considered under positive selection. The number of codons per cistrons was determined and used to normalize the abundance of positive and negative selection.

### 4.8. Geographical Origin and Host Range

For each orthotospovirus accession, the corresponding GenBank file was parsed to get its country of origin and source host, using a custom bash script. Results were captures into a text file used to determine the frequency of each virus species, geographical origin and host.

### 4.9. Intergenic Region Sequence and Structural Alignment

For segment S, 141 TSWV isolates, intergenic region sequences were extracted by parsing of GenBank file using a custom python script. The resulting file was used to align with LocRNA, an RNA alignment tool with the percent identity of >95% and *p*-value < 0.01. The output file (aln) was imported into Geneious to visualize the consensus and identity plots.

To determine the genetic relationship between the hairpin sequences across TSWV isolates, a phylogenetic tree was constructed using MAFFT version 7.3 [78]. A tree based progressive method was utilized to generate Multiple Sequence Alignment (MSA) using the fasta file as input. Using the lowest Bayseian Information Criterion (BIC) [81], the best fit nucleotide substitution model was estimated under Smart Model selection module in PhyML version 3.0 (Methodes et Algorithmes pour la Bioinformatique, Universite de Montpellier, Montpellier, France) [82]. Subsequently, a Maximum likelihood phylogenetic tree was drawn and the output file was used as XML file to generate circular annotated phylogram using GraPhlAn (Centre for Integrative Biology, University of Trento, Trentino, Italy) [83]. Gbmung, a small C-based program (https://github.com/sdwfrost/gbmunge) was used to create annotation file (as txt format) which is required as a supporting file to create annotated phylogram. For each orthotospovirus accession, corresponding GenBank file was parsed into tab-separated metadata containing source information of country of origin and host and plotted as outer ring over the phylogram.

### 4.10. Intergenic Region Hairpin RNA Structure Modelling

The secondary structure of the hairpin in the intergenic region of segment S was modeled using RNAfold in the viennaRNA package [84]. Two separate files, each containing American or Eurasian isolates, were generated by parsing the GenBank file and the intergenic region RNA sequences extracted using a custom python script. RNA folding was modeled using the thermodynamics-based free energy minimization algorithm. For the minimum free energy (MFE) structure, we used the default parameters. RNA consensus structure prediction was done with RNAalifold [85].

### 4.11. Characterization of Polymorphisms in the TSWV Intergenic Region

The GenBank files of 141 TSWV isolates were separated based on geographical origin into to Eurasian and American isolates. Sequence files described in the section above, containing the intergenic region sequences, were used. For each group of isolates, sequences were aligned separately using MAFFT alignment tool [78]. Mafft derived alignment file was used to identify SNPs from both set via SNP-sites version 2.4.1 [79]. For each substitution, the details were obtained in a variant call format (VCF). With respect to consensus sequence generated, SNPs were characterized for their types and frequency was captured. Utilizing VCFtools [80], SNP density was obtained in a 50-nt window of the length of hairpin in each clade and plotted in MS Excel. A comparison was made between to Eurasian and American isolates, using Venny program to identify common, and specific polymorphisms. A heatmap was obtained in MS Excel based on the frequency of each polymorphism.

### 4.12. Effect of Mutations on Hairpin Secondary Structure

RNAmute [43] and RNAsnp [44] were used to detect sites with substitutions. The sites called by both programs were considered significant. Wild-type and mutant sequences were compared based on the predicted minimum free energy (MFE) structures by tree-edit distance and hamming distances on base pairs considering the whole dot-plot or base pairing probabilities plot into account. Large differences between MFE’s indicated a difference in structure. The selection pressure was measured on the RNA secondary structure using SSS-test [46].

## 5. Conclusions

The genome of orthotospoviruses is highly variable. Segments S and M are more variable than segment L. In segments S and M, the intergenic region is more variable than the open reading frames and forms a hairpin that regulates transcription termination and translation. The hairpin is a dynamic structure with multiple functional shapes. Hyper variation in the intergenic region is a general feature of orthotospoviruses, is under diversifying selection, and may be a major determinant of host and vector adaptation.

## Supplementary Materials

The following are available online at https://www.mdpi.com/2076-0817/9/7/521/s1, Figure S1. Schematic representation of the workflow for the identification of nucleotide and amino acid variation in orthotospoviruses, Figure S2. Phylogeny of the genus Orthotospovirus based on segment M, Figure S3. Phylogeny of the genus Orthotospovirus based on segment S, Figure S4. Genome-wide variation in Capsicum chlorosis virus, Figure S5. Genome-wide variation in Calla lily chlorotic spot virus, Figure S6. Genome-wide variation in Pepper chlorotic spot virus, Figure S7. Genome-wide variation in Groundnut bud necrosis virus, Figure S8. Genome-wide variation in Zucchini lethal chlorosis virus, Figure S9. Genome-wide variation in Groundnut ringspot virus, Figure S10. Genome-wide variation in Hippeastrum chlorotic ringspot virus, Figure S11. Genome-wide variation in Melon yellow spot virus, Figure S12. Genome-wide variation in Impatiens necrotic spot virus, Figure S13. Phylogram of TSWV isolates, Figure S14. Representative structures of the segment S hairpin in TSWV isolates from Eurasia, Figure S15. Representative structures of the segment S hairpin in TSWV isolates from the USA. Table S1. Reference accession by virus species and genomic segment. Only accessions containing full-length segments L, M, or S were included in the analysis.
